# Music therapy in modulating immune responses and enhancing cancer treatment outcomes

**DOI:** 10.3389/fimmu.2025.1639047

**Published:** 2025-07-23

**Authors:** Ye Fu, Ke Wu, Jiajia Zhuang, Yisheng Chen, Lina Jia, Zhiwen Luo, Rong Sun

**Affiliations:** ^1^ Division of Moral and Legal Education, Institute of Marxism, East China University of Science and Technology, Shanghai, China; ^2^ Kangwon National University, Chuncheon, Republic of Korea; ^3^ Ningde Municipal Hospital, Ningde Normal University, Ningde, Fujian, China; ^4^ Department of Orthopaedics, Hebei Medical University, Shijiazhuang, Hebei, China; ^5^ Department of Sports Medicine, Huashan Hospital, Fudan University, Shanghai, China; ^6^ Fudan University–Dr. Kong Joint Laboratory of Sports Medicine, Shanghai, China; ^7^ Department of Orthopaedics, the Second Affiliated Hospital of Jiaxing University (Sports Hospital of Jiaxing), Jiaxing, Zhejiang, China; ^8^ Department of Radiation Oncology, Jinling Hospital, Affiliated Hospital of Medical School, Nanjing University, Nanjing, China

**Keywords:** music therapy, cancer, immune modulation, inflammation, integrative oncology, neuroimmune interaction, tumor microenvironment, single-cell resolution

## Abstract

Music therapy, an intersection of art and science, is gaining recognition as a complementary approach in cancer care. This review systematically explores its mechanisms, applications, and effectiveness, with a focus on its impact on the tumor microenvironment (TME), particularly immune signaling and inflammation at single-cell resolution. Evidence shows that music therapy alleviates psychological symptoms—such as anxiety and depression—and physical discomforts including pain, fatigue, and nausea. Beyond symptomatic relief, it also modulates immune responses, especially in immune cell populations that influence tumor-associated inflammation and cancer progression. Advances in single-cell technologies may begin to explain how music therapy modulates immune signaling pathways within the TME, potentially enhancing treatment efficacy.Despite its non-invasive, cost-effective nature and high patient acceptability, music therapy remains underutilized in oncology. Further large-scale studies are needed to elucidate its molecular mechanisms, refine intervention models, and validate its role in immune modulation. As research advances, music therapy holds promise as a valuable component of integrative oncology, supporting patient recovery and immune homeostasis.

## 1 Introduction

Cancer treatment often leads to significant physical and psychological distress, including anxiety, depression, pain, and fatigue, which greatly reduce quality of life (1). Since emerging in the mid-20th century, music therapy has become widely used in psychiatry, rehabilitation, palliative care, and oncology (2, 3). Facilitated by trained professionals, it includes both active music-making and passive listening to promote emotional and physiological well-being (4).

As a supportive therapy alongside standard cancer treatments, music therapy is increasingly recognized for its benefits. Many colorectal cancer patients, for example, seek complementary therapies, with music therapy ranking among the most preferred. Evidence from randomized controlled trials shows that music-based interventions can significantly reduce anxiety, depression, pain, and fatigue, leading to improved quality of life (5–7).

Emerging research also indicates that music therapy may influence immune function by reducing stress-related neuroendocrine activity, potentially enhancing treatment response. Despite some methodological limitations, consistent findings support its therapeutic value, with high patient acceptability and adherence. Recognizing these benefits, organizations like the International Society of Integrative Oncology recommend its use to manage cancer-related psychological distress (8). While most music therapy currently occurs in clinical settings, expanding access to home-based options may improve continuity of care, patient autonomy, and long-term outcomes.

## 2 Basic forms and mechanisms of music therapy

Music therapy is an evidence based clinical intervention that utilizes music experiences to achieve therapeutic goals (9). In cancer care, it serves both psychosocial and physiological functions, offering tailored support across different stages of diagnosis and treatment (10, 11). This section outlines the primary forms of music therapy and the underlying mechanisms through which it exerts its therapeutic effects.

### 2.1 Forms of music therapy

Music therapy includes two main forms: active and passive. Active Music Therapy involves creating music (e.g., singing or drumming), promoting self-expression and emotional connection—especially useful in group settings. Passive Music Therapy involves listening to music to reduce anxiety and stress, ideal for frail patients or those in medical procedures (12). Both forms can be tailored to patient needs. Sessions typically last 30–60 minutes, 2–3 times per week for 4–8 weeks for best results.

### 2.2 Mechanisms of action in music therapy

The effectiveness of music therapy in cancer care is supported by psychological, physiological, and neurological mechanisms (13). Music helps regulate emotions, lower stress, and support brain function. Pleasant and structured music reduces anxiety, fear, and emotional distress in cancer patients (14, 15). It encourages emotional expression, eases depression, and improves psychological well-being (16). Music also serves as a distraction from pain and negative thoughts, reducing discomfort (17). Group music therapy further improves hope, coping, and treatment outlook by encouraging sharing and emotional connection.

These psychological benefits support music therapy’s role in cancer treatment, where emotional well-being affects treatment success. Physiologically, music influences the autonomic nervous system (ANS) and hormone levels (18). Calm music increases parasympathetic activity, reducing heart rate, blood pressure, and muscle tension (19). Clinical trials show music can increase heart rate variability, indicating relaxation (20). In surgical settings, music can lower inflammation markers like IL-6 and HMGB-1, reducing stress responses (21).

Music also affects stress hormones. It lowers cortisol—often elevated during chemotherapy or surgery—and increases oxytocin, which helps with bonding, pain relief, and emotional comfort (22). Music activates brain reward systems, releasing endorphins and dopamine, which reduce pain and enhance tolerance (23). This matches the gate control theory of pain, where non-painful input like music blocks pain signals. Music therapy, therefore, helps reduce stress, control inflammation, and manage pain (24). Animal studies and tissue analysis have confirmed these effects (25, 26).

These hormonal and chemical effects support better hormone balance, less anxiety, and improved immune stability during cancer treatment. Music activates brain areas involved in emotion and motivation—such as the amygdala, nucleus accumbens, hippocampus, and prefrontal cortex (27, 28). This affects mood, focus, and decision-making. Music also influences the HPA axis, controlling cortisol and enhancing immunity (29). Active participation in music (e.g., singing or rhythm exercises) stimulates the brain and supports neuroplasticity, which is important for long-term or palliative care (30). Advanced techniques like single-cell sequencing and multi-omics suggest music therapy can help modulate immune responses, aiding recovery in advanced cancer (31–34). Combining music therapy with nanotechnology and biomaterials could further improve immune regulation and tissue repair (35–38). The type of music and personal preferences also matter—patient-chosen or culturally familiar music often produces better immune and psychological outcomes (39). For example, it has been linked to lower cortisol and higher natural killer (NK) cell activity.

In summary, the neurobiological effects of music therapy support its growing use in cancer care by improving quality of life, emotional resilience, immune balance, and cognitive health in patients, especially those with advanced-stage cancer.

### 2.3 The modernization of oriental music therapy: five-element music therapy combined with artificial intelligence

Recent advances in artificial intelligence (AI) and wearable technology have helped modernize traditional music therapies like Five-Element Music Therapy, which is based on East Asian Five-Element Theory. AI can personalize music therapy by analyzing various sound elements and adjusting rhythm, pitch, and harmony to match a patient’s emotional and physical state, making treatment more tailored and effective (40). Wearable health devices combined with AI further enhance music therapy by continuously tracking indicators like heart rate, skin conductance, and movement. This real-time data helps adjust the therapy for better results. Studies show that these AI-integrated wearables are well-received by patients, especially those with chronic illnesses, as they support remote monitoring and proactive care (41). This is particularly useful when in-person visits are limited, allowing therapy to adapt as the patient’s condition changes. AI and wearable technology are also being used in other healthcare areas. For example, VR paired with wearables has shown success in reducing pain and improving satisfaction during post-surgery recovery (42). VR music therapy can improve emotional and physical health by creating immersive, calming environments. Patient responses vary based on age, culture, gender, and illness. Younger people may prefer fast rhythms, while older adults respond better to soothing music. Cultural familiarity enhances emotional impact, and different cancer stages require tailored support. AI can use these factors to personalize Five-Element Music Therapy. With AI and wearable tech, music therapy becomes more objective and adaptive, combining traditional methods with modern healthcare. As AI advances, it can make music therapy more personalized and effective, especially for chronic and long-term care.

### 2.4 Home-based music therapy for neurodegenerative disorders

Home-based music therapy has shown effectiveness in managing symptoms of neurodegenerative diseases like dementia and stroke. Recent pilot studies support its feasibility and therapeutic value. In one study, 18 dementia patients and their caregivers took part in weekly Neurologic Music Therapy (NMT) sessions for six weeks. Results showed reduced symptoms like agitation and apathy, with improvements lasting up to 12 weeks, also easing caregiver stress (43). Another pilot study with stroke patients experiencing arm weakness involved twice-weekly music therapy for six weeks. Participants responded well, and the therapy showed promise in aiding motor recovery (44). These findings suggest that home-based music therapy is a practical, patient-centered approach that can support long-term recovery, complement standard care, and improve overall well-being.

## 3 Application of music therapy in different stages of cancer treatment

Due to its flexibility and diverse modalities, music therapy can be integrated across all stages of cancer care to address patients’ evolving physical and psychological needs (45). From preoperative preparation and perioperative periods to chemotherapy, radiotherapy, and palliative care, tailored music interventions have been shown to alleviate emotional distress, modulate physiological stress responses, and improve overall treatment experiences and outcomes (46).

### 3.1 Preoperative relief of anxiety and emotional tension

**Figure 1:**
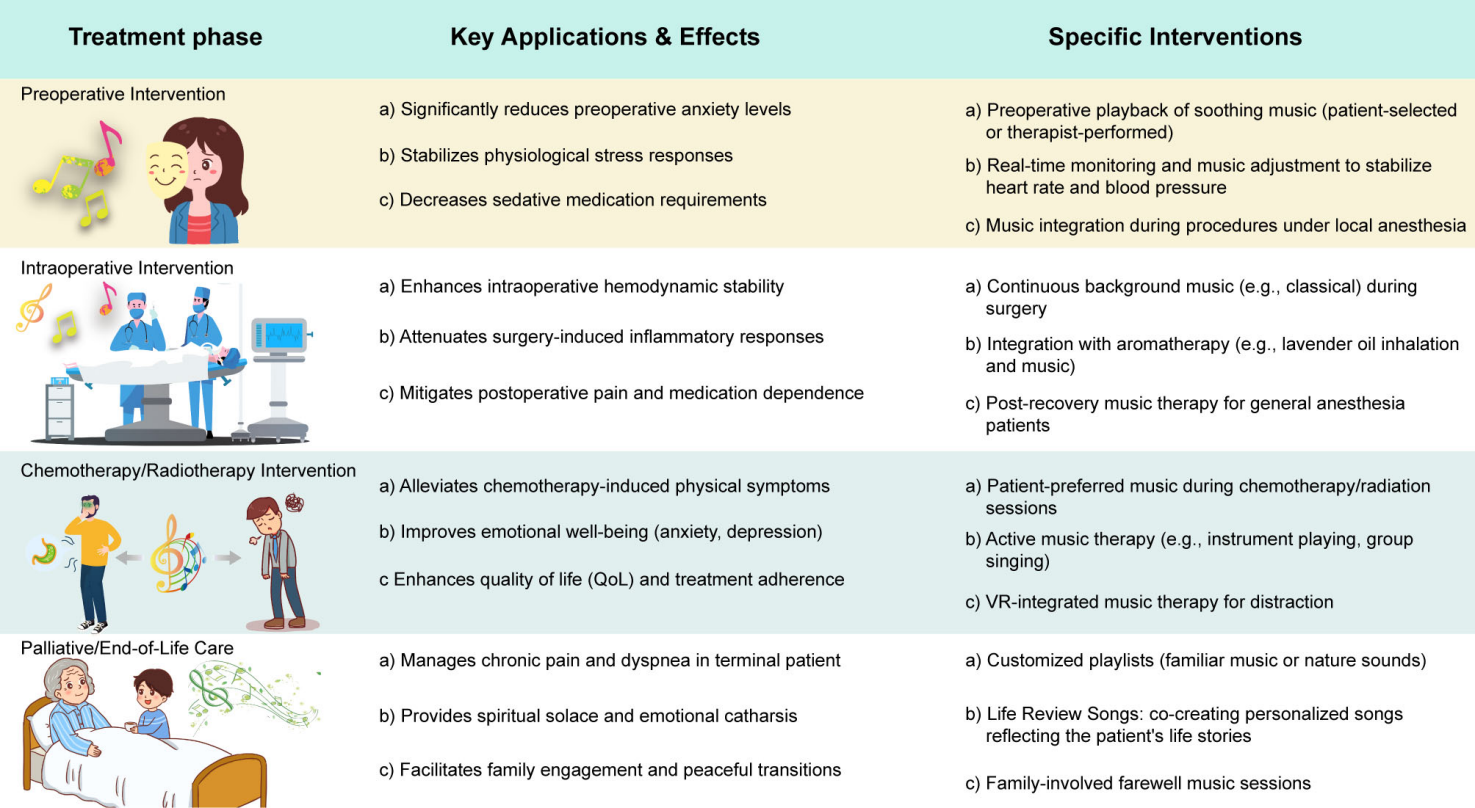
Application of music therapy in various stages of tumor treatment.

Prior to surgery, cancer patients often experience significant anxiety, which may exacerbate pain sensitivity and increase perioperative complications (47). Music therapy effectively addresses these concerns by promoting relaxation and emotional stability. Studies have shown that listening to soothing or self-selected music before surgery reduces anxiety levels and enhances psychological readiness(**Figure 1**) (48, 49). For example, randomized trials involving breast, head and neck cancer patients demonstrate that both live and recorded music significantly decrease preoperative anxiety compared to standard care (50). Music also influences physiological parameters. Patients exposed to calming music exhibit more stable heart rates and blood pressure, and in procedures using local or conscious sedation, music can enhance sedative effects, reducing the need for pharmacologic agents (51). Implementation is straight forward nursing staff may play music in waiting areas, or therapists can provide live sessions. Self-selected music consistently shows greater efficacy, emphasizing the importance of patient preference (52). In summary, preoperative music therapy supports both psychological and physiological stability, potentially improving intraoperative conditions and postoperative recovery.

### 3.2 Intraoperative sedation and decompression

Music therapy during surgery especially under local or regional anesthesia can promote calmness and reduce intraoperative stress (53). For conscious patients, music acts as a distraction from surgical stimuli, enhancing subjective well-being and stabilizing autonomic responses such as heart rate and blood pressure(**Figure 1**) (54, 55). Even under general anesthesia, music has been associated with reduced postoperative pain, anxiety, and analgesic use, suggesting that it modulates stress responses through unconscious pathways (56, 57). Biochemical evidence also supports music’s impact. A study involving breast cancer patients found that intraoperative music significantly reduced pro-inflammatory markers such as IL-6, particularly when combined with aromatherapy. These findings imply a potential role for music in mitigating surgical stress and improving recovery.

In practice, music interventions are non-invasive and do not interfere with surgical procedures (54). Music can be delivered through operating room sound systems or via therapists offering live, adaptive performances. Volume control is critical to maintain communication and patient comfort. For patients under general anesthesia, music contributes to a more serene environment, while for conscious patients, it serves as a vital sensory and psychological support (58).

### 3.3 Benefits in chemotherapy and radiotherapy

Cancer treatments like chemotherapy and radiotherapy often cause distressing side effects (59). Music therapy can help by reducing symptoms, improving emotional well-being, and supporting treatment adherence (60). It has been shown to relieve pain, nausea, and fatigue (**Figure 1**) (3), enhance immune function, and reduce stress, making treatment more tolerable (61, 62). Music promotes relaxation, releases endorphins, and helps balance the immune system, potentially lowering infection risk. Cochrane reviews and bioinformatics studies support its role in symptom management (63). Music therapy also reduces anxiety and depression during prolonged treatment (64), offers emotional support, and may lower the need for medication. Compared to virtual reality, it remains an accessible tool for emotional care (65). By improving both physical and emotional health, music therapy enhances quality of life, increases treatment completion rates, and strengthens patient-provider relationships. In summary, music therapy offers comprehensive support in cancer care, improving outcomes and reducing treatment side effects.

### 3.4 Advantages in palliative and end of life care

In palliative care, the goal shifts to symptom control and quality of life enhancement. Music therapy provides meaningful support for managing physical discomfort and addressing emotional and spiritual needs during this sensitive phase (66).

Music therapy complements pharmacological approaches by promoting relaxation and alleviating symptoms such as pain, fatigue, dyspnea, and insomnia (67, 68). Studies have shown improvements in physiological markers of relaxation and reduced subjective distress in patients receiving music therapy (69). Although some effects, such as pain reduction, may not always reach statistical significance, benefits in fatigue relief and overall comfort are consistently observed.

As patients confront end of life realities, music serves as a medium for emotional expression and spiritual reflection (70). Familiar songs can evoke meaningful memories and foster a sense of peace and connection (71). Therapists often guide patients through life review processes or song composition, enabling expression of love, regrets, and closure (72). These experiences enhance existential well-being and are supported by both qualitative and quantitative research.

Music therapy also benefits families, creating a calm and supportive environment (**Figure 1**). Shared listening or singing can strengthen bonds and offer comfort during final moments. Therapists may facilitate farewell rituals that help patients and families express sentiments and process grief (73). Research shows that families involved in music therapy report lower bereavement distress and a more meaningful end of life experience (74).

In sum, music therapy in palliative and end of life care delivers holistic support—addressing physical symptoms, facilitating emotional peace, and offering spiritual enrichment. It exemplifies the essence of humanistic medicine, affirming dignity and compassion when curative treatments are no longer possible.

## 4 Effects of music therapy in specific types of cancer

### 4.1 Breast cancer and colorectal cancer

Breast cancer patients are a key focus in music therapy research, with benefits observed throughout treatment. During surgery, music therapy reduces anxiety and post-operative pain (75), and studies show it lowers inflammatory markers like IL-6 and HMGB-1, indicating reduced stress (76). Combined with aromatherapy, it has an even greater impact on pain, anxiety, and inflammation (77). During chemotherapy, music therapy continues to support mental and physical well-being (78). An Italian trial found both virtual reality and music therapy improved mood and reduced anxiety, with VR being slightly more effective for anxiety, depression, and fatigue (79). In colorectal cancer, where patients face pain, bowel issues, and emotional strain, music therapy has shown similar benefits. It eases symptoms during surgery, chemotherapy, and palliative care, potentially reducing pain medication use and improving comfort during stoma care (80). These findings reflect wider oncology research supporting music therapy’s role in symptom management and emotional support.

### 4.2 Other types of cancer

Beyond breast and colorectal cancers, music therapy has shown promise in various oncological subgroups (81). In patients with hematologic malignancies undergo hematopoietic stem cell transplantation(HSCT), music therapy has been associated with reduced emotional distress, including tension and depression, and enhanced coping and social interaction. It offers emotional expression and connection, mitigating the isolation commonly experienced during HSCT (82).

In pediatric and adolescent oncology, music therapy is integral due to its interactive nature (83). Studies report improved comfort, reduced procedural fear and pain, and enhanced calmness in hospitalized children. For instance, during lumbar punctures, children receiving music therapy exhibited significantly less pain and anxiety than controls (84). Similarly, live music therapy via closed circuit television during radiotherapy improved treatment compliance and reduced sedation needs (85). These findings highlight music therapy’s value in pediatric oncology care.

While direct research on music therapy in lung, prostate, and brain cancer remains limited, general oncology studies suggest it may alleviate common symptoms such as pain and anxiety (86). Future investigations should address potential differential effects across specific cancer types.

In conclusion, despite varying treatment pathways and clinical needs, music therapy consistently demonstrates positive effects across cancer types. Whether in solid tumors, hematologic malignancies, or pediatric populations, music therapy contributes to pain relief, emotional regulation, and holistic care, thereby supporting its integration into comprehensive oncology treatment frameworks.

## 5 Comparative analysis of music therapy vs. music medicine

Music-based interventions in clinical care include music therapy, delivered by certified therapists, and music medicine, which uses pre-recorded music administered by healthcare staff (87). Both aim to reduce symptoms like pain, anxiety, and stress, but differ in interactivity, personalization, and therapeutic depth. Music therapy is a structured, adaptive process involving active or receptive techniques such as guided listening, singing, or instrument playing (2). Therapists tailor interventions in real time to match patients’ emotional and physiological states, often integrating mindfulness practices and working alongside psychiatrists or psychologists to address complex psychosocial needs (88). This interactive approach can enhance both psychological and physiological outcomes. In contrast, music medicine is passive, simpler, and more scalable, making it useful in resource-limited settings, but less effective for deeper emotional support. Cochrane reviews and other studies show music therapy yields superior outcomes in fatigue, depression, and quality of life due to its relational nature (89). Additionally, music therapy has shown stronger immune-modulating effects, increasing biomarkers like immunoglobulin A (IgA) and NK cell activity, while music medicine mainly reduces cortisol and anxiety. A tiered approach is recommended: music medicine can serve as a broad, accessible option, while music therapy is reserved for patients needing personalized, multidimensional support. Intelligent algorithms and data analysis now support tailored music therapy strategies (90, 91). Furthermore, active participation (e.g., singing or rhythmic activity) often results in greater benefits than passive listening, even within music medicine (92). Both models can be implemented in wards or dedicated therapy rooms. Wards allow flexibility but may require volume controls to avoid disturbing others. Treatment rooms offer controlled settings for advanced sessions, though scheduling may be a limitation. Appointment systems can help manage access. Multidisciplinary collaboration in either setting enhances holistic care. Ultimately, the choice of setting should be based on infrastructure and patient needs to ensure effective, patient-centered delivery.

## 6 Cost effectiveness analysis and practical feasibility

Amid rising healthcare constraints, the widespread adoption of music therapy must consider cost-effectiveness and practicality. Compared to pharmacological or high-tech treatments, music therapy is low-cost, mainly involving therapist salaries and basic equipment (93, 94). Passive music medicine, using playback devices and pre-recorded music, is even more economical (95). Beyond direct costs, it can reduce symptom severity, hospital stays, and medication use, generating indirect economic benefits (96). For example, it lowers postoperative analgesic use and related opioid side effects (97), and reduces the need for sedatives and sleep aids by easing anxiety and depression (98). Music therapy may also modulate immune function, potentially decreasing pharmaceutical reliance (99, 100). It is safe, non-invasive, and well tolerated, with minimal risks compared to drugs. Mild discomfort from unsuitable music can be managed through proper selection. High patient satisfaction and low dropout rates, especially among terminal patients, indicate strong compliance (101, 102). Operational feasibility depends on integration into clinical workflows. While passive music can be delivered by regular staff, formal therapy requires trained professionals (103). Despite current shortages, the number of therapists is growing, and hospitals can employ them or partner with community programs. Successful implementation in some cancer centers demonstrates institutional feasibility. Flexible delivery formats—including bedside sessions, group workshops, concerts, and tele-music therapy—expand access (104). Digital health tools and bioinformatics may enhance treatment strategies (105). A pilot study on virtual mindfulness-based music therapy showed 73% completion without major technical issues (106). Emerging evidence, including single-cell RNA sequencing, suggests music therapy may influence immune signaling pathways, supporting its role in reducing healthcare costs (99). Personalized music based on culture or preference further improves acceptance across diverse populations (107).

In summary, music therapy is a cost-effective, safe, and widely accepted adjunct to cancer care. By integrating professional and passive formats, leveraging digital tools, and considering cultural factors, it can be scaled for broader clinical use.

## 7 Conclusion

**Figure 2:**
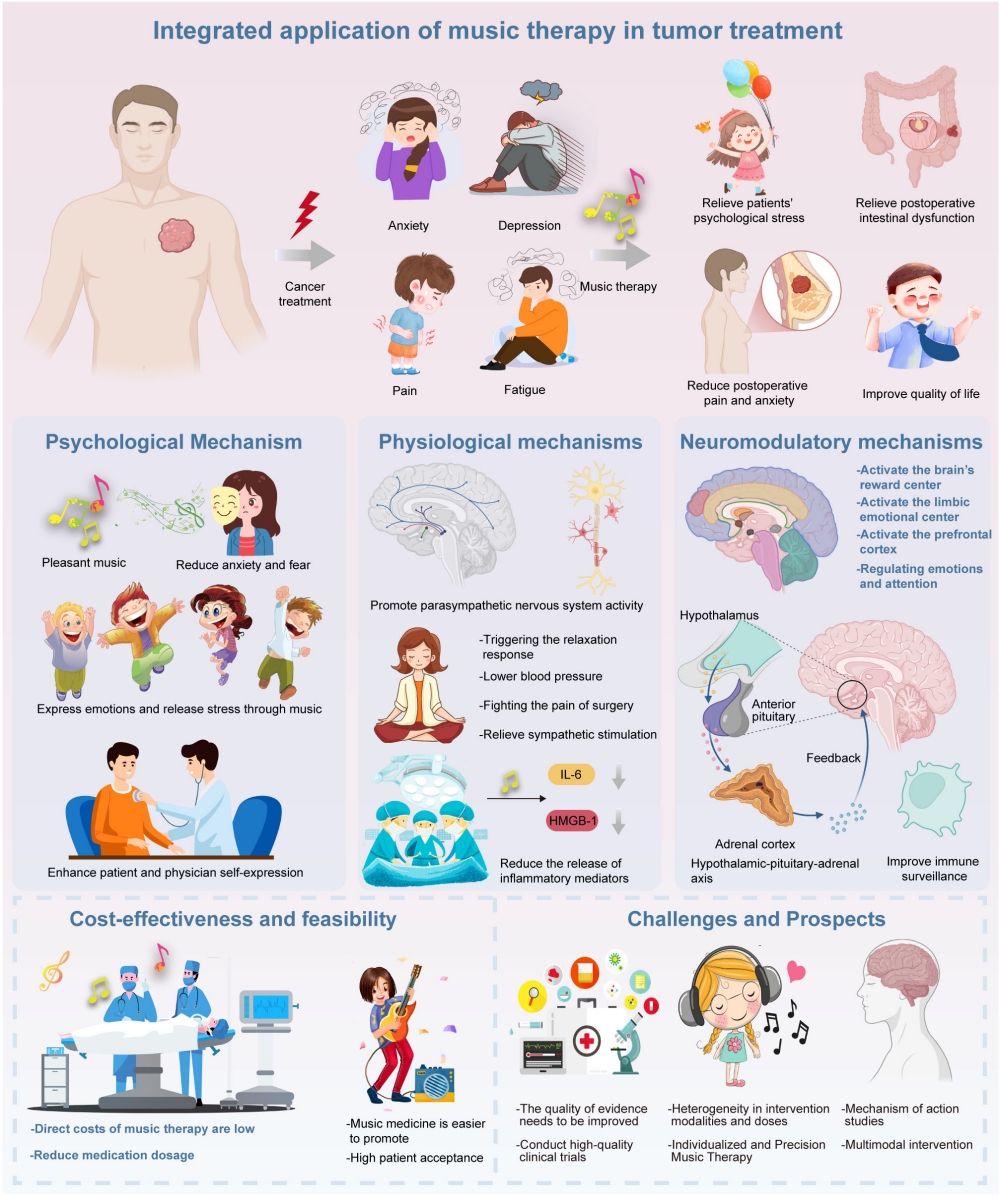
The figure illustrates the therapeutic benefits of music therapy in cancer care, demonstrating its effectiveness in alleviating symptoms like anxiety, depression, pain, and fatigue, thus improving patients’ psychological well-being and overall quality of life. Music therapy promotes emotional expression, reduces anxiety, strengthens patient-provider relationships, and induces relaxation. It also lowers blood pressure, reduces inflammation (e.g., IL-6), and mitigates treatment side effects. Neurologically, music activates brain regions linked to reward and resilience, while enhancing immune surveillance. Music therapy is cost-effective, well-accepted by patients, and can reduce medication use. However, challenges remain, such as the lack of standardized protocols and limited understanding of its biological mechanisms. Future research should focus on large-scale trials and personalized approaches.

Music therapy offers multidimensional benefits across all stages of cancer care, improving psychological symptoms (e.g., anxiety, depression), physical discomfort (e.g., pain, fatigue, nausea), and overall quality of life (**Figure 2**). Despite its high patient acceptability, low cost, and minimal side effects, its integration into oncology is limited by a shortage of trained therapists, inconsistent protocols, and insufficient large-scale evidence. Still, music therapy supports holistic, patient-centered care, reduces pharmacological reliance, enhances treatment adherence, and provides emotional support from diagnosis through end-of-life care. Future research should focus on large-scale RCTs, standardization, exploration of neurobiological mechanisms, and integration with technologies like virtual reality (108). Establishing dedicated services and including training in oncology education will be key to broader implementation.
